# Differential diagnosis of temporal lobe lesions with hyperintense signal on T2-weighted and FLAIR sequences: pictorial essay

**DOI:** 10.1590/0100-3984.2018.0117

**Published:** 2020

**Authors:** Larissa Marques Santana, Eduardo de Jesus Agapito Valadares, Marcos Rosa-Júnior

**Affiliations:** 1 Hospital Universitário Cassiano Antônio de Moraes da Universidade Federal do Espírito Santo (HUCAM/UFES), Vitória, ES, Brazil.

**Keywords:** Temporal lobe, Magnetic resonance imaging, Limbic encephalitis, Neurosyphilis, Herpes simplex, CADASIL, Lobo temporal, Ressonância magnética, Encefalite límbica, Neurossífilis, Herpes simples, CADASIL

## Abstract

Various neuropathologies produce hyperintense signals on T2-weighted or fluid-attenuated inversion recovery sequences of the temporal lobes. Recognition of the distribution pattern and associated findings may narrow the spectrum of differential diagnoses or suggest a specific disease. This pictorial essay aims to illustrate the relatively common diseases that affect the temporal lobe, such as herpes simplex encephalitis, neurosyphilis, limbic encephalitis, postictal edema, neoplasia, and multiple sclerosis, as well as those that are less common, such as myotonic dystrophy type 1, CADASIL, and CARASIL, together with the particularities of each entity.

## Abstract (pt)

Diversas neuropatologias apresentam hipersinal em T2 ou FLAIR nos lobos temporais, porém, o reconhecimento do padrão de distribuição e achados associados podem estreitar o espectro de diagnósticos diferenciais ou sugerir uma doença específica. Este ensaio iconográfico visa demonstrar doenças que acometem o lobo temporal e que são relativamente comuns no dia-a-dia dos radiologistas, como encefalite herpética, neurossífilis, encefalite límbica, edema pós-crise convulsiva, glioma e esclerose múltipla, e outras nem tão comuns como distrofia miotônica tipo I, CADASIL e CARASIL, atentando para as particularidades de cada entidade que auxiliam no diagnóstico.

## INTRODUCTION

Although many diseases manifest with lesions in the temporal lobes, there are few that are restricted to such lesions or that present temporal predominance. The pattern of lesion distribution and the associated imaging findings should become better known, because they can narrow the spectrum of differential diagnoses and suggest a specific pathology.

The objective of this pictorial essay was to demonstrate the magnetic resonance imaging (MRI) changes in diseases that manifest with hyperintense signals on T2-weighted or fast fluid-attenuated inversion recovery (FLAIR) sequences of the temporal lobe.

## HERPES SIMPLEX ENCEPHALITIS

Herpes simplex encephalitis is caused by the *Herpes simplex* virus and is the most common cause of fulminant necrotizing viral encephalitis. The clinical presentation can include fever, headache, focal neurological deficits, convulsions, and an altered state of consciousness^(1)^.

**Figure 1:**
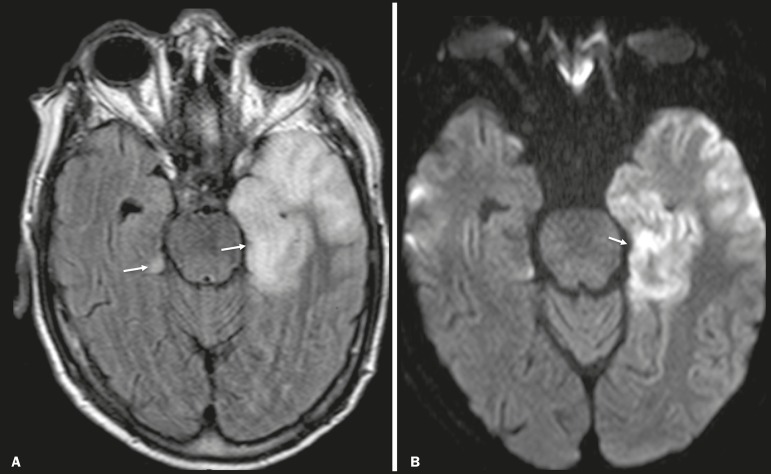
Herpes simplex encephalitis. Axial FLAIR sequence (**A**) and DWI sequence (**B**), showing a hyperintense signal that is diffuse in the left temporal lobe and less pronounced in the right temporal lobe, with mesial involvement and restricted diffusion.

In cases of herpes simplex encephalitis, areas of edema with hyperintense signals in the white and gray matter can be seen in T2-weighted and FLAIR MRI sequences. The temporal involvement can be bilateral, and it is usually asymmetric. Contrast enhancement can be absent in the initial phase, and, on diffusion-weighted imaging (DWI), restricted diffusion due to cytotoxic edema is not uncommon, typically being associated with irreversible neuronal damage^(1)^. Hemorrhaging can be detected in a susceptibility-weighted imaging sequence. The main diagnostic clue is involvement of the mesial portions of the temporal lobes (Figure 1).

## NEUROSYPHILIS

The causative agent of neurosyphilis is *Treponema pallidum*. Like that of a systemic infection, the clinical presentation of cerebral involvement can vary greatly and depends on the stage of the disease. In neurosyphilis, the presentation also depends on the area of the central nervous system (CNS) that is affected. In clinical practice, neurosyphilis is commonly seen in HIV-infected and other immunocompromised patients^(2)^.

**Figure 2:**
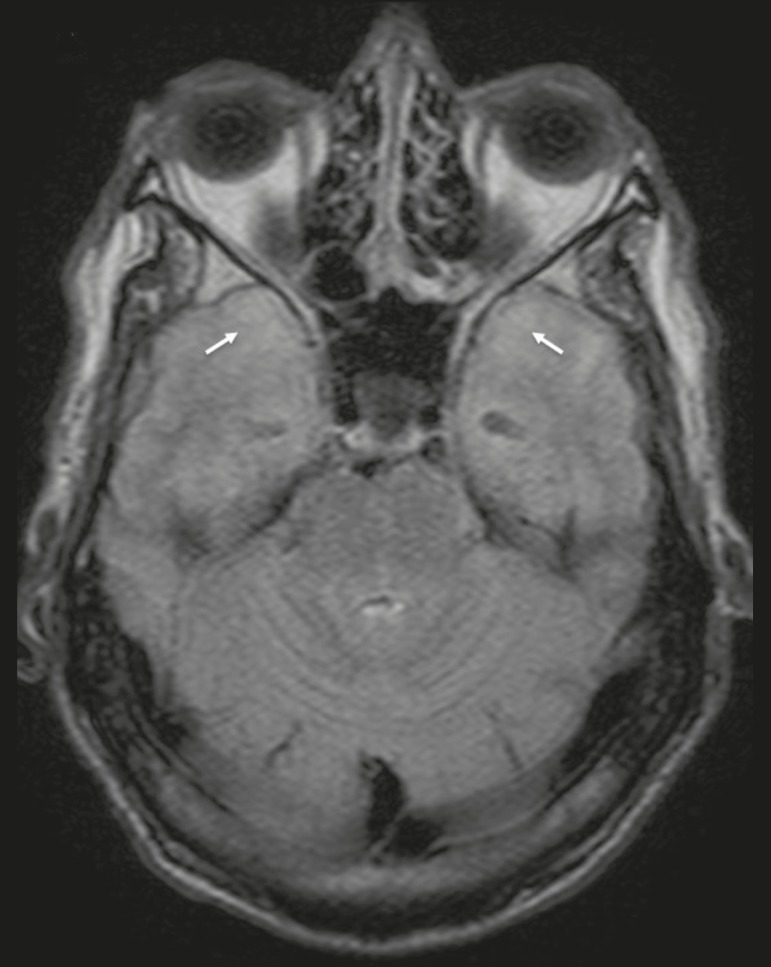
Neurosyphilis. Axial FLAIR sequence showing a hyperintense signal in the white matter of the temporal lobes.

Despite the wide spectrum of manifestations on MRI, syphilitic lesions may present contrast enhancement and hyperintense signals in FLAIR sequences, usually without restricted diffusion on DWI. In cases of acute syphilitic meningitis, the image characteristics are indistinguishable from those of herpes simplex encephalitis, making the biochemical correlation essential^(2)^. There are reports of meningeal, meningovascular, ocular, and parenchymal involvement (Figure 2), as well as of involvement of the calvaria^(2,3)^.

## LIMBIC ENCEPHALITIS

Limbic encephalitis is an inflammatory process mediated by antibodies that typically involve the limbic system, although it can also affect the white matter in other areas of the brain, the brainstem, or the basal ganglia. It can have paraneoplastic or non-neoplastic origins, paraneoplastic encephalitis showing little response to immunotherapy^(4)^. Clinical findings range from alterations in short-term memory to alterations in mental state and can include psychiatric symptoms. In cases with a paraneoplastic origin, the most commonly found neoplasm is small cell lung carcinoma^(4)^.

**Figure 3:**
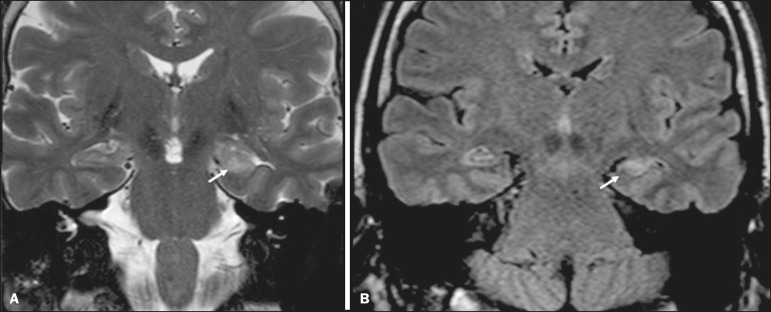
Limbic encephalitis. Coronal T2 sequence (**A**) and FLAIR sequence (**B**), showing a hyperintense signal in the left hippocampus.

On MRI scans of individuals with limbic encephalitis, variable involvement of the temporal lobes can be seen (Figure 3), rarely presenting contrast enhancement or truly restricted diffusion on DWI. Although foci of bleeding are not common, when they are present, other causes are more likely. Up to 25% of cases of limbic encephalitis do not show any alterations on MRI^(4)^.

## MULTIPLE SCLEROSIS

Multiple sclerosis is a demyelinating disease of the CNS. Approximately 90% of lesions are supratentorial, and 10% are infratentorial. Such lesions can involve the deep, juxtacortical white matter, as well as being able to affect the white and gray matter simultaneously. Multiple sclerosis plaques are perpendicular to the lateral ventricles, forming the so-called “Dawson’s fingers”. Lesions in the temporal lobe are usually ovoid and focal with ill-defined margins, tending to converge. The diagnosis is made on the basis of extent of the lesions and of their progression over time^(5)^.

**Figure 4:**
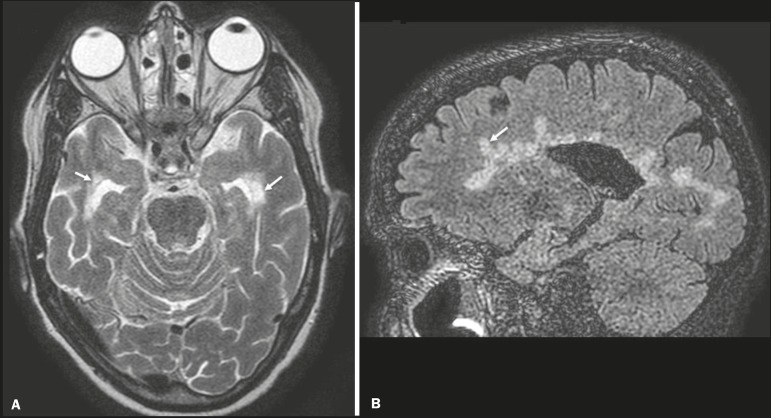
Multiple sclerosis. **A**: Axial T2-weighted sequence showing hyperintense signals in the periventricular and subcortical portions of the temporal lobes. **B**: Sagittal FLAIR sequence showing “Dawson’s fingers”.

Chronic multiple sclerosis lesions present with hypointense signals on unenhanced T1-weighted MRI sequences, whereas active lesions show impregnation on gadolinium contrast-enhanced sequences, can be nodular or annular, and may or may not present restricted diffusion on DWI, which results from intramyelinic edema^(5)^. In the temporal lobe, the involvement of lesions with hyperintense signals in FLAIR sequences occurs predominantly adjacent to the surface of the temporal horns of the lateral ventricles^(5)^, as shown in Figure 4.

### Cerebral autosomal dominant arteriopathy with subcortical infarcts and leukoencephalopathy

Cerebral autosomal dominant arteriopathy with subcortical infarcts and leukoencephalopathy (CADASIL) is a microvasculopathy characterized by recurrent ischemic lesions of the white matter causing vascular dementia in young and middle-aged patients^(6)^. Clinically, it presents as transient ischemic attacks or stroke, although it can also manifest as an intense headache similar to a migraine^(6)^. Histological analysis shows angiopathy of the small- and medium-caliber arteries, with no atherosclerosis or deposition of amyloid protein^(6)^.

**Figure 5:**
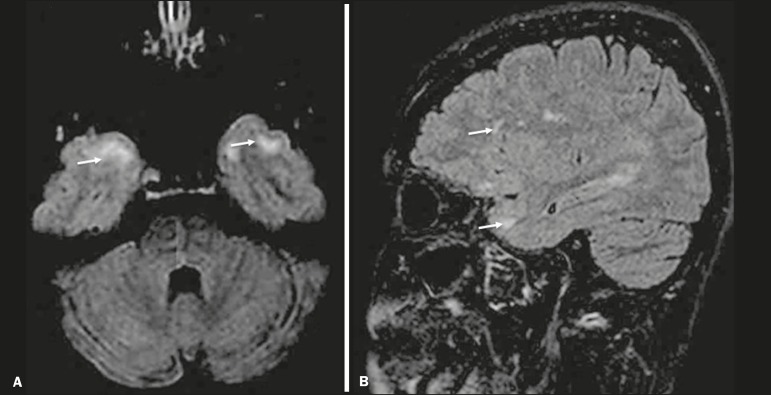
CADASIL. Axial and sagittal FLAIR sequences (A and B, respectively), showing areas of hyperintense signal in the subcortical and anterior portions of the temporal lobes.

In individuals with CADASIL, confluent areas with hyperintense signals can be seen in FLAIR MRI sequences of the white matter and DWI assists in identifying areas of acute and subacute ischemia. At the onset of the disease, the most commonly affected sites are the anterior temporal lobe and the external capsule. In the temporal lobe, the lesions are peripheral, predominantly near the subcortical region. The subcortical occipital, orbitofrontal, and cortical white matter are relatively preserved^(6)^, as shown in Figure 5.

### Cerebral autosomal recessive arteriopathy with subcortical infarcts and leukoencephalopathy

Cerebral autosomal recessive arteriopathy with subcortical infarcts and leukoencephalopathy (CARASIL) is a systemic disease that affects the small blood vessels of the brain stem, hair follicles, and brain. It generally occurs in young adults and has a clinical presentation that is even more severe than that of CADASIL, with early alopecia, vascular dementia, and severe lumbar spondylosis. Histologically, CARASIL is characterized by severe atherosclerosis of the small-caliber arteries^(7)^.

**Figure 6:**
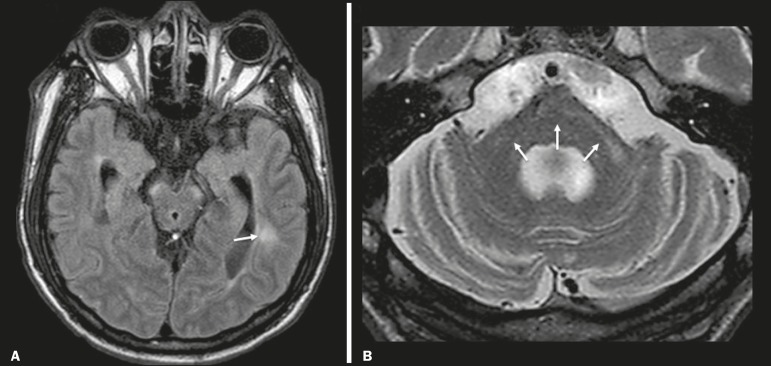
CARASIL. **A**: Axial FLAIR sequence showing a hyperintense signal in the periventricular portion of the left temporal lobe. **B**: Axial T2-weighted sequence showing an arc-shaped hyperintense signal in the pons (“split pons sign”). In a T2*-weighted sequence, there were areas with hemosiderin deposits (not shown).

In individuals with CARASIL, an MRI scan can reveal diffuse leukoencephalopathy with multiple lacunar infarctions. In T2-weighted sequences, diffuse, symmetric lesions with hyperintense signals can be seen in the white matter, typically in the periventricular white matter and deep white matter. As in CADASIL, DWI assists in identifying areas of acute/subacute ischemia. In some patients, anterior involvement of the temporal lobes and external capsule, which is an early sign of CADASIL, can be seen; however, unlike what is seen in CADASIL, the CARASIL lesions are confluent since the initial stage of the disease. A typical sign of this disease in the later stages is the “split pons sign”, consisting of hyperintense lesions in the pons^(7)^, as shown in Figure 6.

## MYOTONIC DYSTROPHY TYPE 1

Also known as Steinert’s disease, myotonic dystrophy type 1 is a dominant autosomal disease with systemic involvement. Clinical manifestations can appear as early as childhood and are characterized by muscle weakness, conduction disturbances, and cataracts^(8)^.

**Figure 7:**
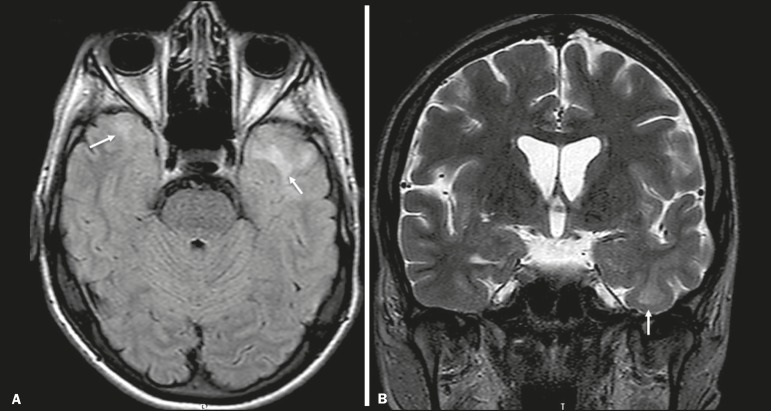
MRI of myotonic dystrophy type 1. **A**: Axial FLAIR sequence showing a hyperintense signal in the white matter of the temporal lobes. **B**: Coronal T2-weighted sequence showing an area of hyperintense signal in the white matter of the temporal lobes. There was no restricted diffusion on DWI (not shown).

On MRI, the alterations caused by myotonic dystrophy type 1 manifest as supratentorial lesions of the white matter and mild cortical atrophy without restricted diffusion on DWI. Although the frontal lobes are most commonly affected, alterations can also occur in the temporo-insular region, which does not occur in myotonic dystrophy type 2. Although MRI is essential for the assessment of these patients, the definitive diagnosis is made by means of genetic testing^(8)^. Figure 7 shows a case of myotonic dystrophy type 1.

## POSTICTAL EDEMA

**Figure 8:**
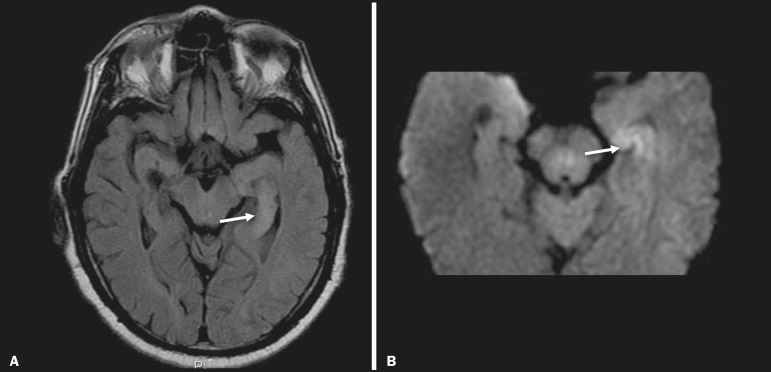
MRI of postictal edema. **A**: Axial FLAIR sequence showing a hyperintense signal in the left hippocampus. **B**: DWI showing restricted diffusion.

In prolonged convulsive crises or in crises repeated at a frequency that does not allow the complete recovery of the patient between crises, an MRI can reveal postictal alterations. The most common such alteration is a hyperintense signal in the cortical gray matter or in the subcortical white matter on T2-weighted and FLAIR sequences, and there can even be restricted diffusion on DWI, with or without contrast enhancement. As depicted in Figure 8, edema often occurs^(9)^.

Similar to what is seen in herpes simplex encephalitis, the sites of involvement in postictal edema vary, including the cortex, subcortical white matter, hippocampi, mesial temporal lobes, and thalamus, the clinical history being essential in these cases. Given the expansile effect, the differential diagnosis should include neoplasia, because it is difficult to rule out an associated tumor when the signal alterations persist over time^(9)^.

## NEOPLASIA

The most common primary CNS tumors are gliomas, which have malignant potential and can be invasive. They arise from glial cells and can be subdivided into astrocytomas, oligodendrogliomas, and ependymomas.

**Figure 9:**
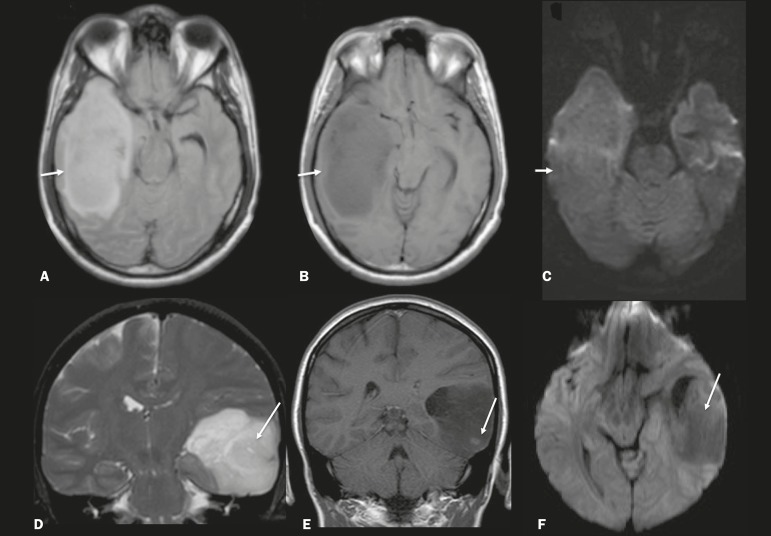
MRI of glioma. **A**: FLAIR sequence showing a hyperintense signal in the cortico-subcortical portion of the right temporal lobe. **B**: Axial T1-weighted sequence showing a hypointense signal with an expansile effect in the right temporal lobe. **C**: DWI showing no restricted diffusion. (**A**, **B**, and **C** all represent a patient with low-grade astrocytoma.) **D**: Coronal T2-weighted sequence showing an expansile lesion, with cortical thickening, in the temporal lobe. **E**: Coronal, contrast-enhanced T1-weighted sequence with a focus of nodular impregnation. **F**: DWI showing no restricted diffusion. (**D**, **E**, and **F** all represent a patient with ganglioglioma.)

Gliomas usually show hypointense or isointense signals in T1-weighted sequences and hyperintense signals in T2-weighted and FLAIR sequences, presenting contrast enhancement or not. The degree to which the diffusion of water molecules is restricted varies according to the cellularity of the tumor^(10)^. Figure 9 shows a glioma with no restricted diffusion. Other types of neoplasms, such as neuroglial tumors, can also affect the mesial temporal lobe and should be included in the differential diagnosis.

## MESIAL TEMPORAL SCLEROSIS

**Figure 10:**
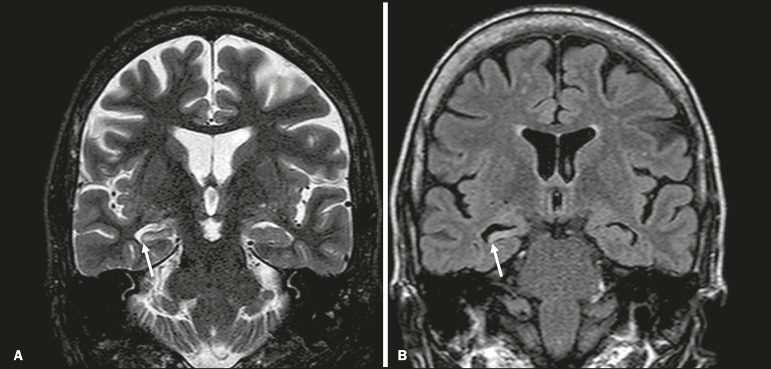
MRI of mesial temporal sclerosis. Coronal T2-weighted and FLAIR sequences (**A** and **B**, respectively), showing a hyperintense signal, atrophy, and right-sided loss of hippocampal digitations in a patient with mesial temporal sclerosis.

Mesial temporal sclerosis is the most common cause of treatment-resistant epilepsy, and it typically manifests with focal seizures without a loss of consciousness. On MRI, it is characterized by hippocampal atrophy, showing a hyperintense signal in T2-weighted and FLAIR sequences, with no restricted diffusion on DWI or morphological alterations of the hippocampus, and is bilateral in up to 10% of cases^(11)^. Figure 10 illustrates these alterations.

## CONCLUSION

It is essential that radiologists know how to identify the diseases that involve the temporal lobe and their particularities, as summarized in Table 1, because such knowledge can facilitate their identification and increase the accuracy of the diagnosis.

**Table 1:** The main characteristics that can facilitate the diagnosis of each of the diseases under study.

Disease	Diffusion	Contrast enhancement	Indicative finding
Herpes simplex encephalitis	Yes/No	Yes / No	Mesial involvement
Limbic encephalitis	Yes/No	Yes / No	Psychiatric symptoms
Neurosyphilis	No	Yes / No	Positive VDRL test result
Multiple sclerosis	Can be restricted at the margins	Only in active lesions	Dawson’s fingers
CADASIL	No	No	Anterior temporal lobe location and external capsule
CARASIL	No	No	Split pons sign
Myotonic dystrophy type 1	No	No	Muscular dystrophy
Postictal edema	Yes	No	Positive clinical history
Invasive glioma	Yes/No	Yes / No	Expansile effect and ill-defined margins
Mesial temporal sclerosis	No	No	Epilepsy, atrophy, and morphological alterations of the hippocampus
